# Poly(glycidyl azide) as Photo-Crosslinker for Polymers

**DOI:** 10.3390/polym14245451

**Published:** 2022-12-13

**Authors:** Xinyan Zhou, Wei Wei, Xiaojian Hou, Gang Tang, Yunjun Luo, Xiaoyu Li

**Affiliations:** 1 Experimental Center of Advanced Materials, School of Materials Science and Engineering, Beijing Institute of Technology, Beijing 100081, China; 2 Science and Technology on Aerospace Chemical Power Laboratory, Hubei Institute of Aerospace Chemical Technology, Xiangyang 441000, China; 3 Key Laboratory of High Energy Density Materials, MOE, Beijing Institute of Technology, Beijing 100081, China

**Keywords:** poly(glycidyl azide), photo-crosslinking, nitrene

## Abstract

Crosslinking polymers to form networks is a universal and routinely applied strategy to improve their stability and endow them with solvent resistance, adhesion properties, etc. However, the chemical crosslinking of common commercial polymers, especially for those without functional groups, cannot be achieved readily. In this study, we utilized low-molecular weight poly(glycidyl azide) (GAP) as polymeric crosslinkers to crosslink various commercial polymers via simple ultraviolet light irradiation. The azide groups were shown to decompose upon photo-irradiation and be converted to highly reactive nitrene species, which are able to insert into carbon-hydrogen bonds and thus crosslink the polymeric matrices. This strategy was demonstrated successfully in several commercial polymers. In particular, it was found that the crosslinking is highly localized, which could endow the polymeric matrices with a decent degree of crosslinking without significantly influencing other properties, suggesting a novel and robust method to crosslink polymeric materials.

## 1. Introduction

The properties of polymeric materials are essentially determined by the chemical structures of the repeat units, and also strongly influenced by their chain architectures, such as linear, branched, brush, network, etc. [1,2,3,4,5]. Even for polymers with the same repeat units, their properties can be largely varied if different chain architectures are adopted [6,7]. In particular, compared to their linear compartment, the polymers with crosslinked network structures usually show significantly better mechanical strengths [8,9,10,11,12], enhanced thermal stability [13,14,15], improved solvent resistance [16,17], etc. Subsequently, chemical crosslinking has been extensively explored and widely applied for the post-polymerization modification of polymer materials. It involves the formation of three-dimensional network structures, causing substantial changes in the material properties. As one of the most typical and well-known examples of crosslinked polymeric systems, vulcanization can convert the fluidic natural poly(isoprene) into strong and elastic rubber, which has been utilized since its discovery in 1890s [18,19].

However, the chemical crosslinking of polymers usually needs to occur through the functional groups on the polymer chains, which are not always readily available. For many commercial polymers, such as polyolefins and polyethers, the lack of reactive moieties leaves very limited choices for crosslinking. For example, the crosslinking of polypropylene usually relies on the formation of macroradicals formed via thermal decomposition of organic peroxide compounds [20], or high-energy gamma or electron beam irradiation [21,22]. However, without the addition of proper coagents, the oxyl radicals generated in peroxide-initiated thermal crosslinking can result in a substantial drop of crystallinity and molecular weight for polypropylene, leading to deterioration of its general properties [23]. Similar detrimental effects can also be observed for the high-dose treatment of high-energy irradiation, and the irradiation source is not easily accessible [24]. A versatile, simple, and robust crosslinking strategy for polymeric materials will be highly desired perpetually.

Meanwhile, compounds bearing multiple precursory azide groups have been applied to produce crosslinked materials, including the photo-crosslinking of intrinsic microporous azide polymer to produce gas separation membrane [25]; photo-crosslinking of azide-containing polymers or difunctional crosslinkers to fabricate a stable active layer for organic light emitting diodes [26], or organic field effect transistors [27], etc. Difunctional azide molecules are also widely used as crosslinkers for photoresists in the photolithography process during the fabrication of microprocessors. Generally, the azide groups can decompose upon heating to above 200 °C or with ultraviolet (UV) irradiation, and convert into nitrene species, which are highly reactive intermediates. They are capable of inserting between C-H to form hydrocarbon amination products, or C=C bonds to form aziridine [28,29,30], and various other bonds [31,32]. However, all these examples of azide-containing crosslinkers require the delicate synthesis of azide-containing compounds or modification of polymers, which is greatly time- and effort-consuming.

**Scheme 1:**
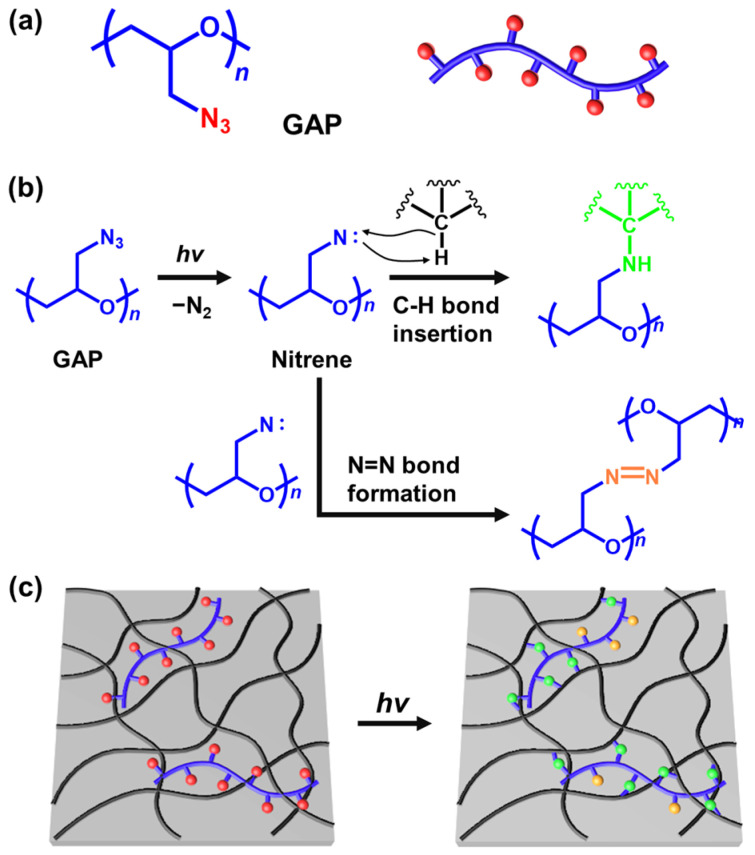
(**a**) Chemical structure and schematic cartoon of GAP; (**b**) The schematic reaction routes for the formation of nitrene from GAP under UV irradiation, the insertion into C-H bond, and the coupling of nitrene to form azo groups; (**c**) Schematic illustration for the crosslinking of polymer/GAP blend film via UV-irradiation.

GAP is a well-known energetic polyether with azide side groups (Scheme 1a) [33,34], and has been commercially available since the 1970s. Due to its high content of azide groups, the enthalpy of formation for GAP can reach 113.8 kJ·mol^−1^ [35], and thus it has been mainly used to produce energetic polyurethanes and applied in energetic composites to enhance the amount of energy released from combustion or explosion. Also because of the azide groups, which are well-known for their ability to undergo “click chemistry” [36,37,38], previous examples of GAP-based crosslinkers were all realized via “click” reactions [39,40,41], and their photo-crosslinking application has barely been explored. In the current study, we report that low-molecular-weight poly(glycidyl azide) (GAP, Scheme 1a, molecular weight 3700 g/mol) could be used as an efficient photo-crosslinker for various polymers. We found that by blending this low-molecular weight GAP homopolymer with various of polymers, the matrices could be crosslinked via the same irradiation-induced formation of nitrene. Owing to the high density of azide groups along the polymer main chain, it can cause highly local crosslinking in polymer matrices (Scheme 1b), which is different from previously reported azide-based crosslinkers. Several typical applications were demonstrated, including significantly increased viscosity of polybutadiene (PB), improved solvent resistance of poly(ethylene glycol) (PEG), and slowed crystallization of poly(vinylidene fluoride) (PVDF). Lastly, we showed that GAP could be applied as photo-initiated adhesives for polymeric plates, suggesting a highly versatile photo-crosslinker for common polymers.

## 2. Materials and Methods

### 2.1. Materials

Poly(glycidyl azide) (GAP, *M*_n_~3700 g/mol) (viscous and brownish) was purchased from Liming Research & Design Institute of Chemical Industry Co., Ltd., Luoyang, China. Polybutadiene (PB) (viscous and colorless) and polyethylene glycol (PEG) (*M*_n_~2000 and 4000 g/mol) (white flake) were purchased from Energy Chemical, Shanghai, China. Polyethylene glycol (PEG) (*M*_n_~20,000 g/mol) (white powder) was purchased from Haian Petrochemical Plant, Nantong, China. Polypropylene (PP) (colorless transparent plastic) plates were purchased from Beijing Tianbao Jinhua Technology and Trade Co., Ltd., Beijing, China. Poly(vinylidene fluoride) (PVDF) (colorless transparent plastic) plates were purchased from Dongguan Yixuan Plastic Materials Co., Ltd., Dongguan City, China. Tetrahydrofuran (THF) (colorless transparent liquid, purity 99.5%) and Dimethyl sulfoxide (DMSO) (colorless transparent liquid, purity 99.9%) was purchased from Tianjin Guangfu Fine Chemical Research Institute, Tianjin, China.

### 2.2. Methods

#### 2.2.1. Photo-Crosslinking of Polymer/GAP Mixture

For PB/GAP mixtures, taking PB/GAP-5 for an example, GAP (0.5 g, 5 wt%) and PB (9.5g, 95 wt%) were added into a 100 mL three-necked flask and heated to 70 °C under vacuum with stirring for 20 min. The mixture was transferred into a quartz bottle and irradiated with ultraviolet light (254 nm) with stirring.

For PEG/GAP mixtures, taking PEG_2k_/GAP-5 for an example, GAP (0.5 g, 5 wt%) and PEG (9.5 g, 95 wt%) were added into a 100 mL three-necked flask and heated to 85 °C under vacuum with stirring for 20 min. The mixture was poured out onto a Teflon plate and irradiated with ultraviolet light (254 nm).

#### 2.2.2. Adhesion between Polymeric Plates with GAP

Four polymeric plates (PP or PVDF) were cut into rectangular plates with a size of 4 cm × 5 cm. A thin layer of GAP was applied on one of the plates, and the other plate was put on the top and pressed tightly to squeeze out excessive GAP. The contact area between the two plates was fixed to be 3 cm × 4 cm. UV irradiation was applied on each side for 60 min.

## 3. Results

**Figure 1:**
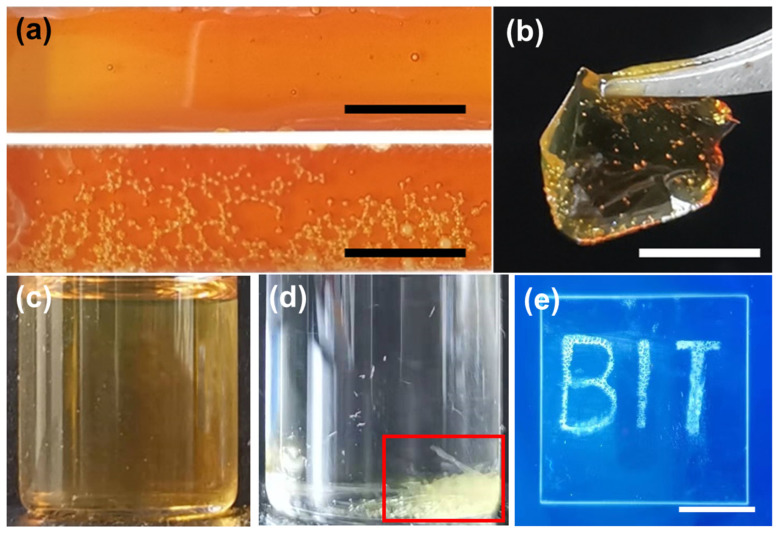
Photographic images of (**a**) GAP samples before (upper) and after (lower) UV-irradiation (254 nm, 20 min); (**b**) a free-standing crosslinked GAP film; (**c**) pristine GAP and (**d**) crosslinked GAP film in THF; (**e**) A pattern of “BIT” letters produced by irradiating the GAP film with a photo-mask. The fragmented GAP film pieces highlighted in red square in (**d**). Scale bars are 1 cm.

Due to its low glass transition temperature (−37 °C, Figure S1), poly(glycidyl azide) (GAP) appeared to be a viscous and brownish yellow liquid at room temperature (21 °C, r.t.). Our study started with the investigation on the photo-crosslinking of GAP homopolymer. The viscous GAP was cast into a polytetrafluoroethylene (PTFE) mold, and was subjected to UV-irradiation at 254 nm (This wavelength was used throughout the paper). As shown in Figure 1a, after 20 min of irradiation in air, a thin film was formed on the top surface of the film, accompanied by the formation of many gas bubbles underneath the film. The resultant film was highly crosslinked and the freestanding film could be picked up with a tweezer (Figure 1b). Moreover, while GAP could be completely dissolved in tetrahydrofuran (THF, Figure 1c), the crosslinked GAP film was insoluble and easily broke into small pieces (Figure 1d). Therefore, a pattern of three letters “BIT” could be produced on quartz plate by simply irradiating the GAP film under a photo-mask (Figure 1e).

These phenomena were attributed to the decomposition of azide groups upon irradiation, releasing nitrogen (N_2_) and yielding highly reactive nitrene (N:) groups. These nitrene groups could insert into C-H bonds, or, alternatively, couple to form azo groups to form a crosslinked network (Scheme 1b,c). However, due to the low molecular weight of GAP and its high crosslinking degree, the film obtained was very fragile, and easily fragmented into small pieces when immersed in THF, as shown in Figure 1d.

**Figure 2:**
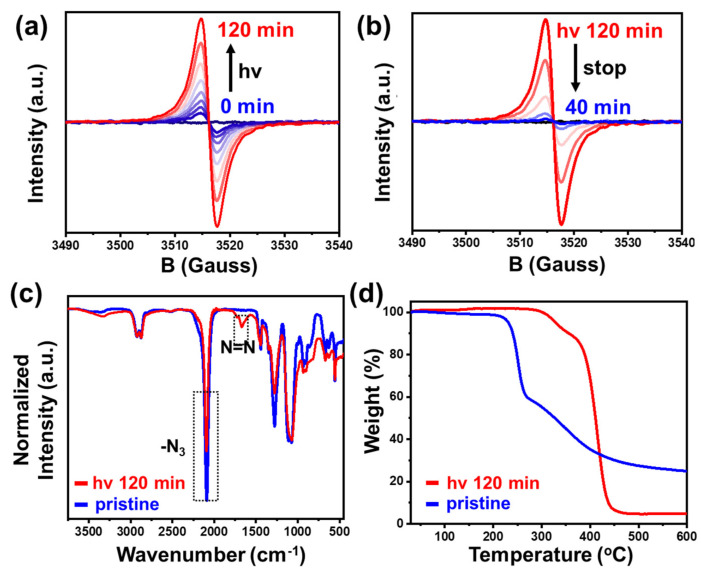
EPR spectrometry of GAP (**a**) with UV-irradiation and (**b**) when the irradiation was stopped in a nitrogen atmosphere; (**c**) FT-IR spectrometry and (**d**) TGA result of GAP samples before (blue) and after (red) UV-irradiation (254 nm, 120 min).

To further verify this explanation, the irradiation-induced crosslinking process was monitored in situ with electron paramagnetic resonance (EPR) spectrometry. As shown in Figure 2a, with continuous UV-irradiation at 254 nm under nitrogen atmosphere, a typical EPR signal for nitrene free radicals appeared after very short-term irradiation [42,43]. The intensity of the signal peak gradually increased, indicating an increase in the amount of nitrene free radicals. Meanwhile, when the irradiation was stopped, the signal peak intensity gradually diminished to zero over time (Figure 2b), possibly because these highly reactive species inserted into C-H bonds on the GAP, or coupled to form azo groups (Scheme 1b). The reduction of signal was much quicker with the presence of air, due to the quenching by oxygen (Figure S2).

Subsequently, the GAP sample before and after UV irradiation was characterized with Fourier-transform infrared spectroscopy (FT-IR). A comparison of the sample before and after UV irradiation for 120 min was shown in Figure 2c. The peak in the 1150 cm^−1^ area shows the vibrations of -C-O-C, proving the break of the GAP chain. The signal peak intensity from the azide group (1350 cm^−1^ and 2100 cm^−1^) was significantly reduced, clearly demonstrating the decomposition of azide groups. Meanwhile, the emergence of the new characteristic peak of azo group (1630 cm^−1^) verified the formation of azo groups in the crosslinked GAP sample. In addition, as shown by the thermogravimetric analysis (TGA) results, the thermal stability of the GAP sample was enhanced significantly after UV irradiation (Figure 2d), due to the removal of labile azide groups and the formation of crosslinked networks.

The results from the above experiments unambiguously showed that, upon UV irradiation, GAP can release nitrogen and generate highly reactive nitrene species, which can form azo groups or insert into C-H bonds. This insertion can occur intramolecularly within the GAP chains, or otherwise intermolecularly between GAP and other polymer chains. Therefore, considering the wide availability of C-H bonds in common commercial polymers, we believe it is possible that GAP can be used as a universal photo-crosslinker.

**Figure 3:**
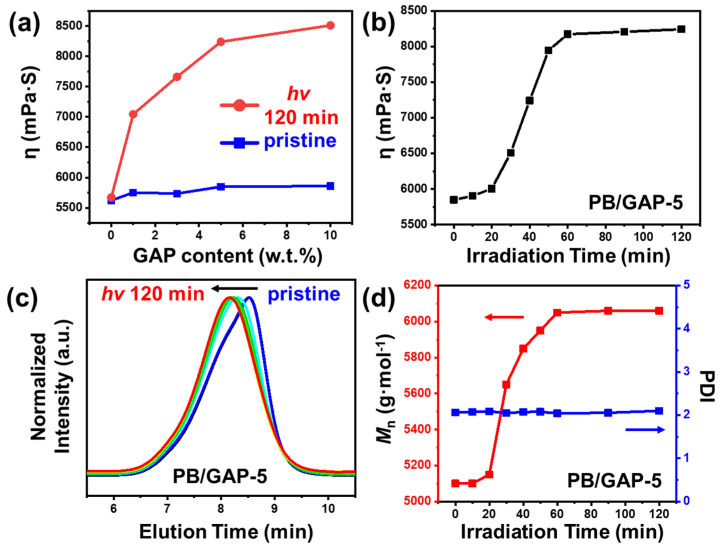
(**a**) Variation of the viscosity of PB/GAP blends before (blue) and after (red) 2 h of UV-irradiation; (**b**) Variation of the viscosity of PB/GAP-5 blend with UV-irradiation time; (**c**) GPC traces of the PB/GAP-5 blend after UV irradiation for different periods of time; (**d**) *M*_n_ and PDI of the PB/GAP-5 blend after UV irradiation for different periods of time.

Our first attempts were to blend GAP with polybutadiene (PB), which also appears to be a viscous but colorless liquid at r.t. Several samples were prepared by blending different amount of GAP into PB, namely PB/GAP-n, with the “n” representing the mass percentage of GAP in the blends. The viscosity of the samples was measured with a rheometer to monitor the change with irradiation time (Figure S3). Since GAP is also a viscous liquid-like material, its content would not significantly influence the viscosity of the PB/GAP blend. However, after the blend was irradiated with 254 nm UV light for 120 min, an obvious increase in viscosity was observed, as shown in Figure 3a. Expectedly, the higher the GAP content was, the higher the viscosity became. The viscosity of the blend PB/GAP-5 was also monitored during the irradiation process (Figure 3b). It was found that, upon irradiation, there was only a slight viscosity increase in the initial 20 min, after which a sudden jump was observed, until it reached a plateau after 60 min. The initial slow increase might be due to the existence of dissolved oxygen inside PB, which quenched nitrene and slowed the crosslinking reaction. After it was consumed, the crosslinking could then process quickly. Despite the existence of double bonds on PB chains, only a slight increase in viscosity was detected after irradiation in our control experiment (Figure S4), suggesting the viscosity increase was indeed induced by the photo-crosslinking of GAP.

However, it was noteworthy that the crosslinking reactions were limited to local regions very close to the GAP polymer chains. Moreover, the content of GAP was 10% at most, which is low comparing to other similar crosslinking systems. Therefore, only a small portion of the PB chains were involved in the crosslinking process. In addition, due to the fact that the PB used in the current study was of low molecular weight (molecular weight ~5000 Dalton), it is expected that only branched PB polymers were formed instead of a uniformly-crosslinked insoluble network. Consequently, their molecular weight could still be characterized via gel-permeation chromatography (GPC). Moreover, considering the low molecular weight and content of GAP, its addition would not significantly influence the molecular weight of the mixture. With UV irradiation, the molecular weight of the mixture increased significantly and a shift in GPC peak was observed (Figure 3c). Similar to the variation trend of viscosity, the molecular weight of PB/GAP-5 also remained almost unchanged over the initial 20 min, which was followed by a sudden jump (Figure 3d). After 60 min of irradiation, the overall molecular weight reached a plateau around 6000 Dalton. This value did not change noticeably even after 120 min of irradiation. Meanwhile, the polydispersity index (PDI) of the polymer remained almost identical throughout the whole process.

**Figure 4:**
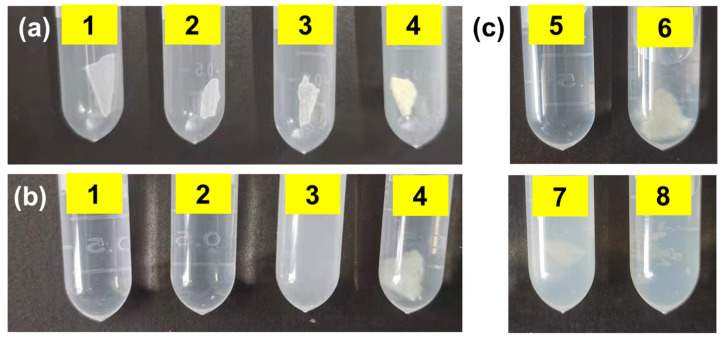
Photographic images of: (1) pure PEG_2k_, (2) PEG_2k_ after irradiation, (3) pristine PEG_2k_/GAP-5 blend, and (4) the PEG_2k_/GAP-5 blend after photo-crosslinking (254 nm, 2 h): (**a**) before immersion in water, and (**b**) immersed in water for 10 min; (**c**) Photographic images of blend samples after photo-crosslinking (254 nm, 2 h): PEG_2K_/GAP-1 (5), PEG_2K_/GAP-5 (6), PEG_4K_/GAP-5 (7) and PEG_20K_/GAP-5 (8) immersed in water for 24 h.

Crosslinking is also a very effective strategy to enhance the solvent resistance of polymers [44,45]. For this demonstration, poly(ethylene glycol) (PEG) with relatively higher molecular weight was chosen as model polymers PEG_2k_, PEG_4k_, and PEG_20k_, where 2k, 4k and 20k represent their molecular weight. PEG was melted upon heating to 80 °C, and GAP was subsequently added to yield PEG/GAP-n blend. When the blends were cooled to r.t., they became solid again, and were subsequently subjected to UV irradiation for 120 min. As shown in Figure 4a, there was no difference visually between the solids of pure PEG_2k_, PEG_2k_ after irradiation, PEG_2k_/GAP blend, and the blend after irradiation (sample #1, 2, 3, 4 in Figure 4a respectively). However, after they were immersed into water for 10 min (Figure 4b), the pristine and irradiated PEG_2k_ solids (sample 1 and 2 in Figure 4b) were completely dissolved, and due to the low GAP content, the PEG_2k_/GAP-5 blend without irradiation could be dispersed and formed a slightly turbid solution (sample 3 in Figure 4b). Meanwhile, after the 120 min of irradiation, the PEG_2k_/GAP-5 blend solid remained clearly visible in water after 10 min, as shown in (sample 4 in Figure 4b). The solvent resistance of other samples were shown in Figure S5.

Besides the irradiation time, the crosslinking effect was also influenced by the content of GAP and molecular weight of PEG. It was found that, while the crosslinked PEG_2K_/GAP-1 (sample 5 in Figure 4c) would be completely dissolved, with higher GAP content (sample 6 in Figure 4c), the crosslinked PEG_2K_/GAP-5 would remain solid even after immersion in water for 24 h. Meanwhile, with the same GAP content and irradiation time, PEG_4K_/GAP-5 (sample 7 in Figure 4c) would only be partially dissolved, but PEG_20K_/GAP-5 (sample 8 in Figure 4c) would be mostly dispersed into a turbid solution.

These results could be further quantified by measuring the weight of PEG/GAP solid before and after immersion in water and calculating the percentage of remaining solid. As shown in Table 1, when the GAP content gradually increased, the resultant PEG_2k_/GAP turned from completely soluble in water (PEG_2k_/GAP-1) to partially soluble, and the insoluble content increased from 26.5% (PEG_2k_/GAP-3) to 36.9% (PEG_2k_/GAP-5). Meanwhile, with increasing molecular weight of PEG, the insoluble content decreased from 36.9% for PEG_2k_/GAP-5 to 24.9% for PEG_4k_/GAP-5, and decreased further to 17.0% for PEG_20k_/GAP-5.

Increasing GAP content could cause higher crosslinking density, easily leading to higher insoluble content. However, it is not obvious why longer PEG chains yielded lower insoluble content. Since the GAP content was kept the same in these samples, theoretically, the crosslinking density should be more or less the same for all these three samples. The melting enthalpy values of the PEG_2k_/GAP-5 was determined to be −153.21 J/g from their differential scanning calorimetry (DSC) curves (Table 2), almost identical to that of pure PEG_2k_ (−159.44 J/g), suggesting negligible influence from the addition of GAP to the crystalline lattice of PEG. Moreover, even after UV irradiation for 120 min, the crosslinked PEG_2k_/GAP-5 showed a very similar value of melting enthalpy (−157.78 J/g). Consequently, a plausible hypothesis could be made here that the GAP chains in the mixture were excluded from the crystalline lattice during the crystallization process and mostly located in between the crystalline grains. Therefore, when the GAP chains were irradiated, the crosslinking mostly occurred between crystalline domains, instead of uniformly throughout the whole polymer matrix. Consequently, when longer PEG chains were applied, larger crystalline domains were formed and the crosslinking would be even less uniform, leading to lower content of insoluble crosslinked product.

**Figure 5:**
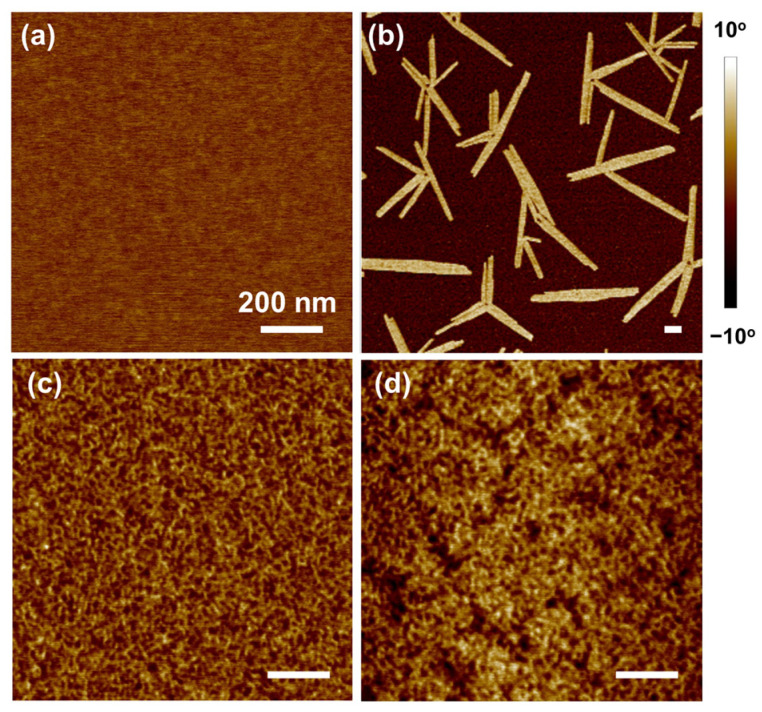
AFM phase images of the films cast from the DMSO solution of PVDF/GAP blend (weight percentage of GAP = 10%): (**a**) as-cast film; (**b**) film was annealed with saturated DMSO vapor for 2 h; (**c**) film was crosslinked via UV-irradiation (254 nm, 2 h) and then (**d**) annealed with saturated DMSO vapor for 2 h.

Subsequently, it should be reasonable to expect that by blending a small amount of GAP with crystalline polymers, it could be used to stabilize the phase-segregated domains and crystalline grains simply via UV irradiation, of which the sizes are highly important for their properties and strongly influence the device performance [46,47]. To verify this, films were cast via spin-coating from the dimethyl sulfoxide (DMSO) solution of GAP and poly(vinylidene fluoride) (PVDF) with a GAP content of 10 wt.%. The as-cast film showed no obvious phase segregation between the two polymers (Figure 5a), due to the low content of GAP and also no crystallization of PVDF. However, after the film was subjected to saturated DMSO vapor for solvent annealing for 2 h, very clear crystal prisms from PVDF were observed (Figure 5b). In sharp contrast, after the film was subjected to UV irradiation for 2 h, clear phase segregation with a domain size around only 10 nm was observed from the Atomic Force Microscopy (AFM) phase image (Figure 5c), which probably could be attributed to the gentle annealing by the heat generated during irradiation. These domains did not further increase in size even after solvent annealing with saturated DMSO vapor for 2 h (Figure 5d). These findings strongly demonstrated our hypothesis that GAP can stabilize the crystalline domains via simple UV crosslinking.

It was noteworthy that, for previous reports on difunctional azide-based or similar crosslinkers, the crosslinking occurred uniformly within the polymeric matrices and formed three-dimensional network structures, usually resulting in much higher glass transition temperature (*T_g_*) and melting temperature (*T_m_*). However, in the current case, the azide groups were concentrated along the GAP polymer chains, and therefore the crosslinking was highly localized. This resulted in a very high degree of crosslinking within very local and sparsely distributed domains, and would not significantly change *T_g_* and *T_m_* of the polymeric matrices. Specifically, the *T_g_* of the PB/GAP-5, and the *T_m_* of the PEG_2K_/GAP-5, after photo-crosslinking for 2 h, continued to be −27.8 °C and 54.4 °C, respectively, almost the same as those without the addition of GAP and crosslinking (−27.8 for PB and 55.0 for PEG) (Table 2 and Figure S6). Therefore, despite their increased molecular weight, they showed improved solvent resistance. After crosslinking, their general properties were not significantly altered, which will be desired for practical applications.

**Figure 6:**
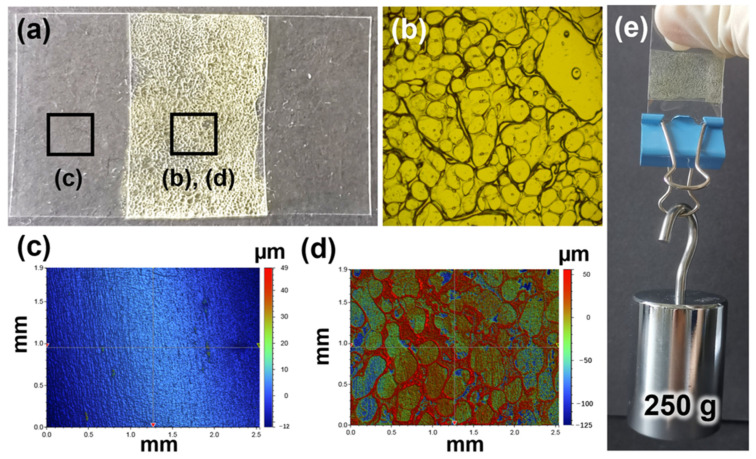
(**a**) Photographic image of the PP plates adhered by crosslinked GAP; (**b**) Optical microscopic image of the adhered region; Optical profile topography images of (**c**) unadhered region and (**d**) adhesion region; (**e**) Photographic image of the 250 g weight lifted by two adhered PP plates.

Moreover, another typical application of crosslinkers could be adhesives between polymer substrates [48,49]. In particular, this nitrene-radical-based crosslinker will be applicable to polymers without functional groups. For example, adhesion of low-surface-energy plastics such as polypropylene (PP) is an important problem in manufacturing. For a proof of concept, a very thin layer of GAP was applied between two PP plates for photo-crosslinking. Ideally, a monolayer of GAP should be applied between the two plates to allow the GAP chains to contact both surfaces. Due to the intrinsic surface roughness of these PP plates, an excessive amount of GAP was applied initially between the two plates, which was subsequently squeezed out by force to keep only the necessary amount of GAP. Consequently, the two pieces were adhered together after 254 nm UV irradiation for 2 h, and many gas bubbles were generated after irradiation (Figure 6a,b). A comparison of the optical profile topographic images of the crosslinked and the non-crosslinked regions showed a very strong contrast: the non-crosslinked region appeared to be very smooth (Figure 6c), but the crosslinked region appeared to be quite rough (Figure 6d). Despite these bubbles and much reduced contact area between the two plates, the adhesion was still strong and the adhered pieces were able to lift a weight of 250 g, which was over 30,000 times of the weight of GAP used, as shown in Figure 6e.

Theoretically, this strategy should be applicable to various other commonly used polymers without functional groups, and even fluorinated polymers, which are of very low surface tension. Typically, a thin layer of GAP was applied between two PVDF plates, which were pressed tightly and irradiated at 254 nm for 2 h. These two plates were strongly adhered together, with similar phenomenon of bubble formation. Interestingly, the tear resistance value from adhered PP plates was determined to be 30.2 kPa, and 28.8 kPa for adhered PVDF plates (Figure S7). These two values were very close, probably due to the fact that the adhesion was mainly through the insertion of nitrene and formation of covalent connections.

## 4. Conclusions

In this paper, we reported the investigation of the application of poly(glycidyl azide) as a photo-crosslinker for polymers. It was demonstrated that UV irradiation at 254 nm can convert the azide groups into nitrene, with the release of nitrogen gas. These highly reactive nitrene species can insert into C-H bonds in various polymers to form three-dimensional networks. Several typical applications were demonstrated, including the significantly increased viscosity of polybutadiene (PB), improved solvent resistance of poly(ethylene glycol) (PEG), and slowed crystallization of poly(vinylidene fluoride) (PVDF). Moreover, this GAP polymer could be used as adhesives to allow even polymer substrates with very low surface tension to be adhered together. Differently from other similar systems, besides the improvement brought by the crosslinking, the general properties of these polymers were not significantly changed, due to the highly localized crosslinking. This work provides an effective strategy for utilizing GAP in the crosslinking field, and may open a new route for the crosslinking of commercial polymeric materials.

## Figures and Tables

**Table 1:** Results of solubility tests of PEG/GAP samples after UV irradiation.

Sample	Solution in 10 min	m_pristine_ (mg)	m_insoluble_ (mg)	Insoluble Content (wt.%)
PEG_2k_/GAP-1	Yes	50.2	0.0	0.0
PEG_2k_/GAP-3	No	49.8	13.2	26.5
PEG_2k_/GAP-5	No	50.4	18.6	36.9
PEG_4k_/GAP-5	No	49.8	12.4	24.9
PEG_20k_/GAP-5	No	50.5	8.6	17.0

**Table 2:** DSC results of PEG and PB samples before and after crosslinking for 2 h.

Sample	Melting Enthalpy (J/g)	*T_m_* (°C)	*T_g_* (°C)
PEG_2k_	−159.4	55.0	N.A.
PEG_2k_ UV	−169.7	54.8	N.A.
PEG_2k_/GAP-5 UV	−157.8	54.4	N.A.
PB	N.A.	N.A.	−27.8
PB UV	N.A.	N.A.	−29.2
PB/GAP-5 UV	N.A.	N.A.	−27.8

## Data Availability

The data presented in this study are available on request from the corresponding author.

## Supplementary Materials

The following supporting information can be downloaded at: https://www.mdpi.com/article/10.3390/polym14245451/s1, Figure S1: DSC curve of GAP; Figure S2: EPR spectra of GAP samples after irradiation at 254 nm for 120 min and then exposed to air; Figure S3: Comparisons of the viscosity of PB/GAP blends before (black) and after (red) 2 h of UV-irradiation at 254 nm; Figure S4: Comparisons of the viscosity of pure PB before (black) and after (red) 2 h of UV-irradiation; Figure S5: Photographic images of pure PEG (1,5,9,13), PEG after irradiation (2,6,10,14), PEG/GAP mixture (3,7,11,15), PEG/GAP mixture after irradiation (4,8,12,16) after they were immersed in water for 10 min: (a) PEG_2k_ and PEG_2k_/GAP-1; (b) PEG_2k_ and PEG_2k_/GAP-3; (c) PEG_4k_ and PEG_4k_/GAP-5; (d) PEG_20k_ and PEG_20k_/GAP-5; Figure S6: DSC curves of the (a) PEG_2k_ and PEG_2k_/GAP-5 samples, and (b) PB and PB/GAP-5samples; Figure S7: Tear resistance curves of the PP (red) and PVDF (black) plates after adhesion with GAP and UV-irradiation (254 nm, 2 h).
