# Supplementary material for: Poly(glycidyl azide) as Photo-Crosslinker for Polymers
Source: Polymers (Basel). 2022 Dec 13;14(24):5451. doi: 10.3390/polym14245451 (PMC9787972; doi:10.3390/polym14245451)
Supplement: Supplementary file 1 [file polymers-14-05451-s001.zip › polymers-2061907-supplementary.pdf]

# Supporting Information

# Poly(Glycidyl Azide) as Photo-Crosslinker for Polymers

**Xinyan Zhou [1], Wei Wei [2], Xiaojian Hou [1], Gang Tang [1], Yunjun Luo [1,2,*] and Xiaoyu Li [1,2,*]**

[1] Experimental Center of Advanced Materials, School of Materials Science and Engineering, Beijing Institute of Technology, Beijing 100081, China
[2] Science and Technology on Aerospace Chemical Power Laboratory, Hubei Institute of Aerospace Chemical Technology, Xiangyang 441000, China
[3] Key Laboratory of High Energy Density Materials, MOE, Beijing Institute of Technology, Beijing 100081, China
* Correspondence: yjluo@bit.edu.cn (Y.L.); xiaoyuli@bit.edu.cn (X.L.)

## Techniques

### Fourier transform-infrared (FT-IR) spectroscopy

The FT-IR spectrum was recorded with a Thermo Scientific Nicolet iS50 system within a range of 400 ~ 4000 $cm^{-1}$ with a resolution of 4 $cm^{-1}$ and averaging 32 scans for each spectrum.

### Electron Paramagnetic Resonance (EPR)

The test was carried out with 254 nm ultraviolet irradiation by A300-10/12 (Bruker Physik-AG) in nitrogen and air respectively.

### Rheometer

The viscosity was tested at 25 °C by a MCR92 rheometer produced by Anton Paar, setting the shear rate at 0.1~100 1/s.

### Gel permeation chromatography (GPC)

The molecular weights and polydispersity indices of the samples were tested at 25 °C with GPC LC-20A, (Shimadzu Co. Ltd), using THF as the mobile phase at a flow rate of 1.0 mL $min^{-1}$. In addition, the raw data were calibrated with polystyrene standards.

### Thermogravimetric analysis (TGA)

TGA analysis were performed on the METTLER Instrument TGASTAR system at heating ramp of 10 °C $min^{-1}$ from 30 °C to 600 °C with the flow rate of nitrogen for 40 mL $min^{-1}$. For each test, 2.00 mg of sample was used.

### Differential scanning calorimetric analysis (DSC)

DSC thermograms were tested by the DSC1 (Mettler Toledo) at a heating rate of 10 K $min^{-1}$ from -80 °C to 150 °C with the flow rate of nitrogen for 40 mL $min^{-1}$. For each test, 2.00 mg of sample was used.

### Atomic force microscope (AFM)

Tapping mode AFM was performed with the Brooke Dimension Fast Scan Atomic Force Microscope. For each test, samples were spin-coated on an aluminum sheet with carbon tap to tested.

### Optical microscope

Optical microscopic characterization was carried out using a Leica DM2500P Optical Profiler and was observed at 40x magnification.

**Three-dimensional optical profilometer**

Optical profilometer characterizations were tested by the Contour GT-X, a next-generation white light interferometer that combines advanced 64-bit multi-core manipulation and analytical processing software. It is the most advanced 3D optical surface profilometer system in years.

**Mechanical property tests**

For mechanical test, samples were cut into cut into rectangular plates with size of 4 cm x 5 cm. A thin layer of GAP was applied in on one of the plates, and the other plates was put on the top and pressed tightly to squeeze out excessive GAP. The contact area between two plates were fixed to be 3 cm x 4 cm, and to be test at r.t. with a loading rate of 20 mm $min^{-1}$ on HZ-1004A universal testing machine.

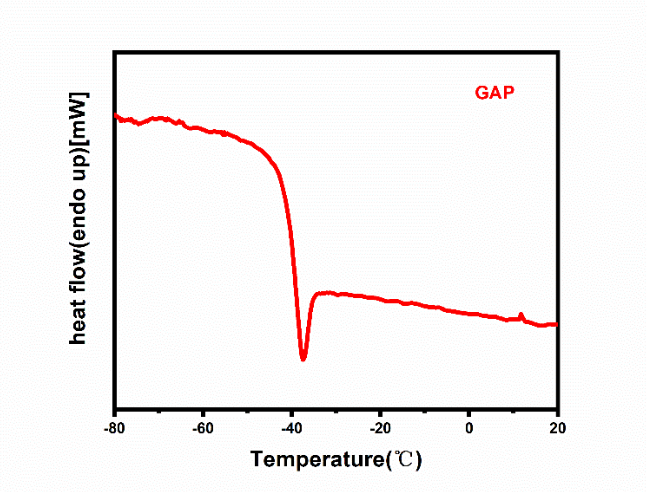


**Figure S1.** DSC curve of GAP.

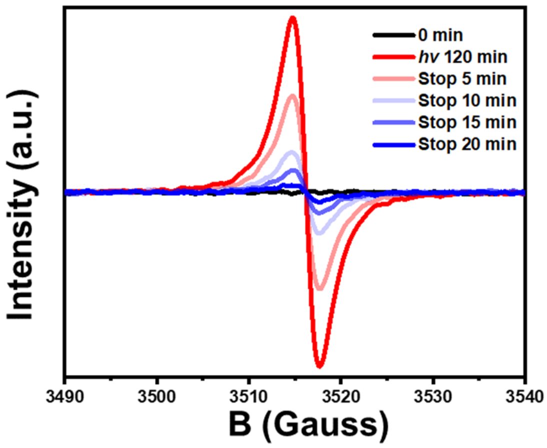


**Figure S2.** EPR spectra of GAP samples after irradiation at 254 nm for 120 min and then exposed to air.

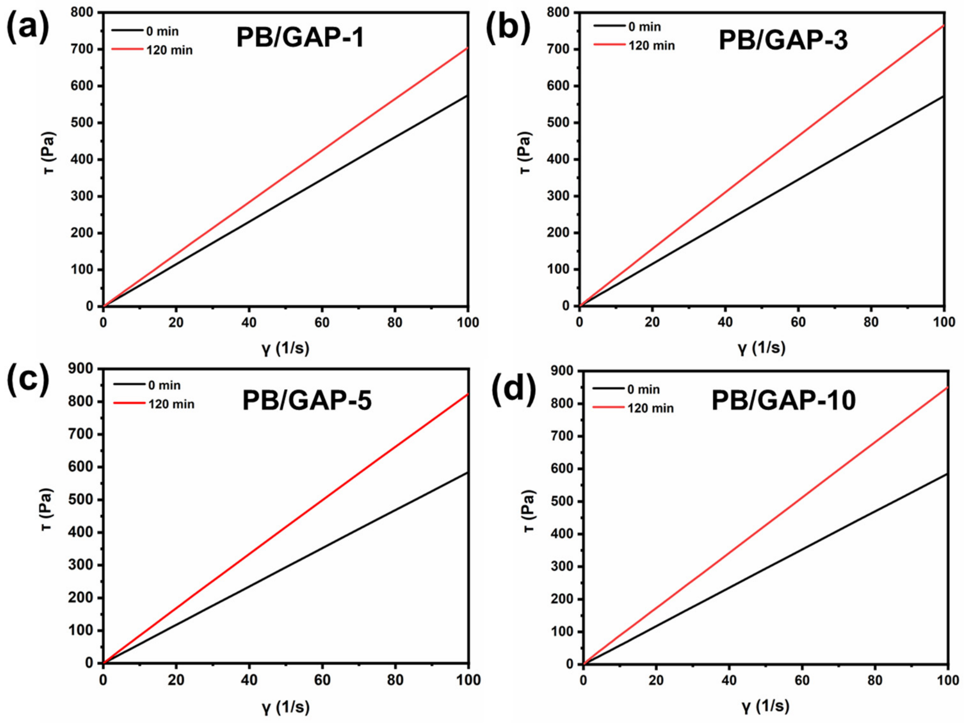


**Figure S3.** Comparisons of the viscosity of PB/GAP blends before (black) and after (red) 2 h of UV-irradiation at 254 nm.

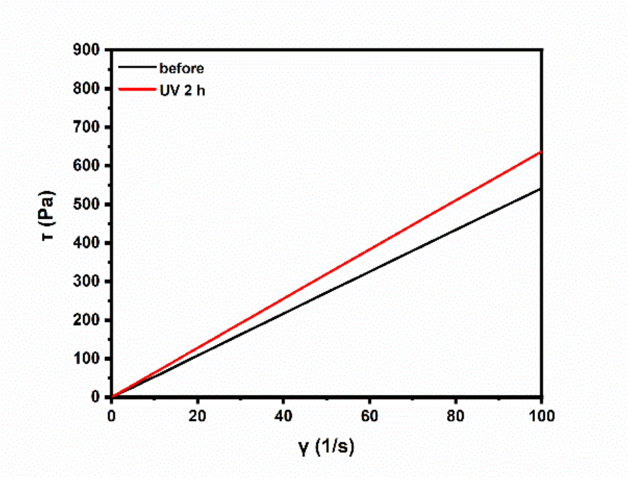


**Figure S4.** Comparisons of the viscosity of pure PB before (black) and after (red) 2 h of UV-irradiation.

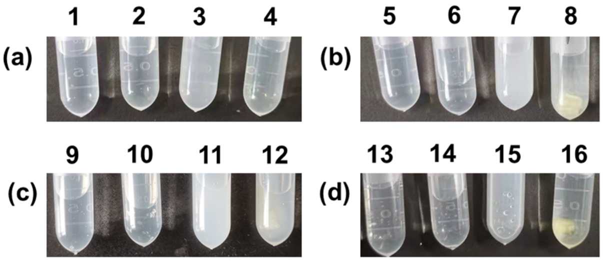


**Figure S5.** Photographic images of pure PEG (1, 5, 9, 13), PEG after irradiation (2, 6, 10, 14), PEG/GAP mixture (3, 7, 11, 15), PEG/GAP mixture after irradiation (4, 8, 12, 16) after they were immersed in water for 10 min: (a) $PEG_{2k}$ and $PEG_{2k}$/GAP-1; (b) $PEG_{2k}$ and $PEG_{2k}$/GAP-3; (c) $PEG_{4k}$ and $PEG_{4k}$/GAP-5; (d) $PEG_{20k}$ and $PEG_{20k}$/GAP-5.

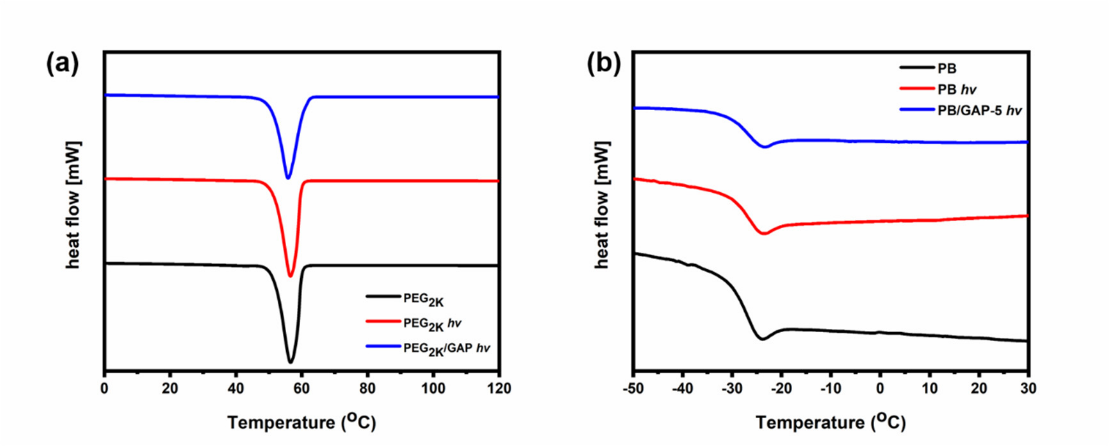


**Figure S6.** DSC curves of the (a) $PEG_{2k}$ and $PEG_{2k}$/GAP-5 samples, and (b) PB and PB/GAP-5samples.

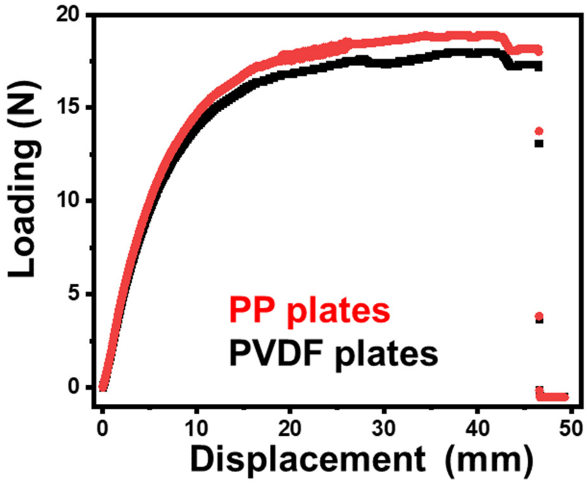


**Figure S7.** Tear resistance curves of the PP (red) and PVDF (black) plates after adhesion with GAP and UV-irradiation (254 nm, 2 h).
